# Multiple linear B-cell epitopes of classical swine fever virus glycoprotein E2 expressed in *E.coli *as multiple epitope vaccine induces a protective immune response

**DOI:** 10.1186/1743-422X-8-378

**Published:** 2011-07-30

**Authors:** Bin Zhou, Ke Liu, Yan Jiang, Jian-Chao Wei, Pu-Yan Chen

**Affiliations:** 1 Key Laboratory of Animal Diseases Diagnosis and Immunology, Ministry of Agriculture, Nanjing Agricultural University, Nanjing 210095, China; 2 Jiangsu Entry-Exit Inspection and Quarantine Bureau, Nanjing 210001, China

**Keywords:** Classical swine fever virus (CSFV), Glycoprotein E2, Linear B-cell epitope, Multiple epitope vaccine (MEV)

## Abstract

Classical swine fever is a highly contagious disease of swine caused by classical swine fever virus, an OIE list A pathogen. Epitope-based vaccines is one of the current focuses in the development of new vaccines against classical swine fever virus (CSFV). Two B-cell linear epitopes rE2-ba from the E2 glycoprotein of CSFV, rE2-a (CFRREKPFPHRMDCVTTTVENED, aa844-865) and rE2-b (CKEDYRYAISSTNEIGLLGAGGLT, aa693-716), were constructed and heterologously expressed in *Escherichia coli *as multiple epitope vaccine. Fifteen 6-week-old specified-pathogen-free (SPF) piglets were intramuscularly immunized with epitopes twice at 2-week intervals. All epitope-vaccinated pigs could mount an anamnestic response after booster vaccination with neutralizing antibody titers ranging from 1:16 to 1:256. At this time, the pigs were subjected to challenge infection with a dose of 1 × 10^6 ^TCID_50 _virulent CSFV strain. After challenge infection, all of the rE2-ba-immunized pigs were alive and without symptoms or signs of CSF. In contrast, the control pigs continuously exhibited signs of CSF and had to be euthanized because of severe clinical symptoms at 5 days post challenge infection. The data from in vivo experiments shown that the multiple epitope rE2-ba shown a greater protection (similar to that of HCLV vaccine) than that of mono-epitope peptide(rE2-a or rE2-b). Therefore, The results demonstrated that this multiple epitope peptide expressed in a prokaryotic system can be used as a potential DIVA (differentiating infected from vaccinated animals) vaccine. The E.coli-expressed E2 multiple B-cell linear epitopes retains correct immunogenicity and is able to induce a protective immune response against CSFV infection.

## 1. Introduction

Classical swine fever (CSF) or hog cholera is a highly infectious viral disease included in the list of diseases notifiable to the OIE (http://www.oie.int). It affects domestic and wild pigs and is considered to be one of the most devastating diseases for the pig industry throughout the world from both the economic and sanitary point of view [1,2]. CSF virus (CSFV), the etiological agent of CSF, is an icosahedral and enveloped positive strand RNA virus that belongs to the Pestivirus genus of the Flaviviridae family [3-5]. Pestivirus RNA contains a single large open reading frame (ORF) flanked by two untranslated regions (UTRs). The ORF encodes a polyprotein of about 3900 amino acids that in infected cells is processed by cellular as well as viral proteases to yield four structural (C, Erns, E1, E2) and eight nonstructural proteins (Npro, P70, NS2, NS3, NS4A, NS4B, NS5A, NS5B) [6-8].

E2 is the essential protein in virus replication and infextation, and it is also the major immunogenic protein that is responsible for inducing neutralizing antibodies and eliciting protective immunity against CSFV [9-12], which has been the main component in the design of CSFV DIVA vaccines [13,14]. Previous studies shown that the N terminus of CSFV E2 has four antigenic domains (A-D), with three subdomains (A1-A3) in domain A. Subdomain A1 and domain B and C are major neutralizing determinants [15,16]. Based on previous study, the neutralizing epitopes corresponding to different regions of the A or BC domains of E2 were proposed and used as vaccines against CSFV in either mono-or multi-peptide formulations [17-20]. Some mixtures of linear peptides have been reported to induce CSFV-specific neutralizing antibodies as well as protection against CSFV challenge infection. However, in most cases they failed to confer complete protection from clinical signs upon viral challenge. On the other hand, little information is available on the effect of these peptide vaccines on the levels of viremia and virus shedding [21,22].

In the present study, two commercially synthesized peptides, which covered the antigenic domain B/C (aa693-716) and A (aa844-865) on envelope glycoprotein E2, induced CSFV-specific neutralizing antibodies and provided pigs with complete or partial protection against CSFV [21,23]. However, it was not reported that these two new epitopes or combination epitope were expressed by prokaryotic system or eukaryotic system to be developed as epitope-based vaccines. Therefore, two B-cell linear epitopes, rE2-a (CFRREKPFPHRMDCVTTTVENED, aa844-865) and rE2-b (CKEDYRYAISSTNEIGLLGAGGLT, aa693-716), were constructed and heterologously expressed in *E.coli *as multiple epitope vaccine(MEV). The results suggested that the E.coil-expressed epitope rE2-ba induced immunoregulation, similar to that of attenuated virus vaccine(HCLV). It is expected that the E.coli-expressed MEV may replace the synthetic peptides and be mass produced for inoculation of large numbers of pigs.

## 2. Materials and methods

### 2.1 Construction of recombinant expression plasmids

E2-a (aa844-865) gene and E2-b (aa693-716) gene were amplified from the plasmid pMD18-T-E2, using two pairs of primers 1a,2a and 1b,2b respectively(Table 1). The amplified E2-a DNA and E2-b DNA were purified from gel and cloned into the pMD18-T vector (Takara Bio., Dalian, China) by the T/A cloning strategy to generate the recombinant plasmid pT-E2-a and pT-E2-b. E2-a DNA fragment was cloned into the SacI and Hind III sites of the plasmid pET-32a(+)(Novagen, Darmstadt, Germany) to construct pET-E2-a. E2-b DNA fragment was cloned into the BamH I and Sac I sites to construct pET-E2-b. Gene E2-b and E2-a were alternately cloned into the BamHI, SacI and HindIII sites to construct a fusion E2-ba DNA. Three resultant constructs were verified by enzyme digestion and DNA sequencing.

**Table 1:** Primers used in this study.

Primer	Oligonucleotides (5' - 3')	Description
1A 2A	GTAGAGCTC(*Sac *I)TTCAGGAGAGACAAGC CGCAAGCTT(*Hind *III)TTAATCTTCATTTTCCAC	aa844~aa865
1B 2B	CGGGGATCC(*BamH *I)TGCAAGGAAGATTACAG AAAGAGCTC(*Sac *I)AGTGAGACCTCCCGCCC	aa693~aa716

### 2.2 Expression and purification of linear B-cell epitope in *E. coli*

Recombinant protein expression was obtained in *E. coli *BL21 grown at 37°C in 50 mL LB medium containing 100 μg/mL ampicillin to an OD_600 _of 0.6. The culture was adjusted to 1 mM IPTG and incubated for a further 14 h. Cells were harvested by centrifugation (6 k rpm,10 min), washed twice with phosphate-buffered saline (PBS), resuspended in PBS, and finally sonicated on ice (8 × for 30 s). The lysate was centrifuged for 15 min at 10 k rpm at 4°C, and the polypeptide composition of the recovered pellet and the supernatant was analyzed by SDS-PAGE [24,25]. As the three recombinant proteins appeared to be present in inclusion bodies, a further lysis step was introduced (8 M urea overnight). Following this, the pellet was centrifuged for 15 min at 10 k rpm at 4°C, and the supernatant was purified on an Ni-affinity chromatography column (Amersham Bioscience HiTrap chelating HP5 mL × 1 column). The purification steps were performed according to the instructions. Only freshly purified proteins were used (with adjuvant) to injected into pigs. Protein concentration was determined by the Bradford method with BSA (bovine serum albumin) as a standard [26].

### 2.3 Western blot analysis

rE2-ba, rE2-a and rE2-b proteins were treated in equal volumes of the unreduced 2 × SDS/PAGE sample buffer (125 mM Tris-Cl [pH 6.8], 20% glycerol, 4% SDS, 0.25% bromophenol blue). As a reducing agent, β-mercaptoethanol was added to the final concentration of 5%. Proteins were separated by 12% SDS-PAGE and transferred by electroblotting onto PolyScreen PVDF transfer membrane (NEN) using semi-dry transfer cell (Bio-Rad) according to the manufacturer's manual [27]. The membrane was then sequentially treated with blocking solution (PBS containing 5% non-fat skim milk), with 200-fold dilution of rabbit hyperimmunity serum specific to CSFV E2 (a generous gift from Prof. Xinglong Yu, Hunan Agricultural University, China), and with 3,000-fold dilution of anti-rabbit IgG goat antibody conjugated to horseradish peroxidase (Cell Signaling TECHNOLOGY). Finally, the membrane was soaked in DAB Reagents (Boshide Biotech Co, Wuhan, China) for signal development.

### 2.4 Immunization and challenge of pigs

**Figure 1:**
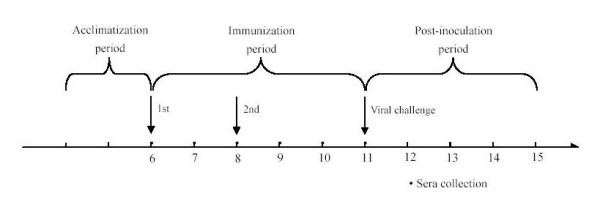
**Vaccination schedule and viral challenge period of in vivo experiment**. Five-weeks-old pigs were acclimatized for 1 weeks and were given a first followed by a second immunization. Pigs immunized were inoculated intranasally with a lethal dose of CSFV on day 21 after the second immunization. Blood samples were collected once weekly from week 7. The health of each pig was assessed twice daily.

Twenty-five 5-week-old piglets with negative CSFV antibody titers, which were purchased from the Animal Disease Centre, Nanjing Agricultural University, were randomly divided into five groups of five each. The pigs were acclimatized for 1 weeks, during which their environmental and body temperatures were measured once daily. After acclimatization, the average temperature of pig's rectal was 39.0 ± 0.5°C. Group A, B and C were repectively inoculated with purified rE2-a, rE2-b, and rE2-ba using ISA 206 VG adjuvant(Seppic, France). The group D was immunized with a commercial vaccine (HCLV) as positive control and the group E was immunized with PBS as negative control. The pigs were inoculated with 500 μg of each peptide for the first and second immunization at an interval of 2 weeks. The immunization procedure is described by Table 2. In 3rd week after the second immunization, all pigs were intranasally inoculated (mimicking natural infection) with a 10^6 ^TCID_50 _dose of virulent CSFV Shimen strain(obtained from the National Institute of Veterinary Drug Control, Beijing, China; GenBank accession number: AF092448). The ambient temperature, rectal temperature, symptoms (petechial hemorrhage, diarrhea and endothelial damage) and food intake were recorded every day during the experiment (Figure 1). Environmental temperature was 24~28°C, and there was no apparent indicator of its relationship to pig body temperature.

**Table 2:** Animal grouping and immunizing dose.

Groups	Pigs	Protein/vaccine name	Description	Dose
A (1-5)	5	rE2-a	aa844~aa865	500 μg/swine
B (6-10)	5	rE2-b	aa693~aa716	500 μg/swine
C (11-15)	5	rE2-ba	aa693~aa716 & aa844~aa865	500 μg/swine
D (16-20)	5	HCLV	positive control	1 dose/swine
E (21-25)	5	PBS	negative control	1 ml/swine

Twenty-five pigs were assigned randomly to five groups. Each group was immunized by intramuscular (i.m.) injection at the midpoint of the left thigh muscle. rE2-a, rE2-b, rE2-ba, HCLV and PBS were administrated to groups A, B, C, D and E, respectively.

### 2.5 Cytokine quantified by ELISA

One week after the second boost vaccination, the presence of cytokine IL-4, IL-10 and IFN-γ in serum samples from vaccinated pigs were determined using commercially available swine cytokine ELISA kits (Jingmei Corporation, Shengzhen, China), following the manufacturer's instructions. In each assay, a control recombinant swine cytokine was diluted over the recommended detection range to generate a standard curve. Sample concentrations were interpolated from the standard curve.

### 2.6 Serological examination

Serum samples from immunized pigs were collected by jugular venipuncture every week after immunization and post-challenge. Each sample was heated to inactivate at 56°C for 30 min and then subjected to detection by neutralization peroxidase-linked assay (NPLA) [28]. All sera duplicate and in serially two-fold dilutions and the titers were determined as the reciprocal dilution of serum that neutralized 100TCID_50 _of strain Shimen in 50% of the culture replicates [29].

### 2.7 Statistical analysis

All values were expressed as the mean ± SD. The ANOVA (F test) was used to determine the significance of differences among the groups. P values < 0.05 were considered statistically significant.

## 3. Results

### 3.1 Expression and Identification of multiple linear B-cell epitopes

**Figure 2:**
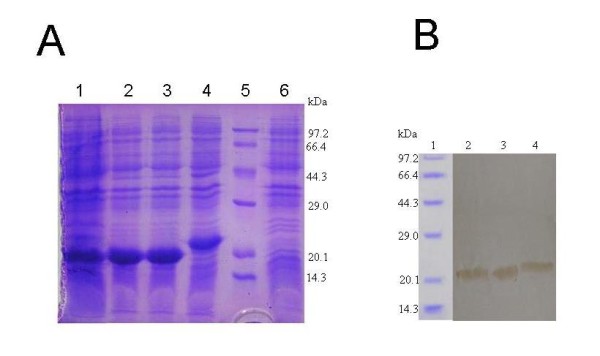
**SDS-PAGE of recombinant proteins and Western blotting**. A: SDS-PAGE of rE2-a, rE2-b and rE2-ba. Lane 1, lysates of the induced E. coli BL21(DE3) pET-32a(+); lane 2-4, lysates of the induced BL21(DE3) pET-rE2-a, BL21(DE3) pET-rE2-b and BL21 (DE3) pET-rE2-ba; lane 5, protein molecular weight markers; lane 6, lysates of the non-induced BL21(DE3) pET-32a(+). B: Western blotting of rE2-a, rE2-b and rE2-ba. Lane 1, protein molecular weight markers; lane 2-4, rE2-a, rE2-b; and rE2-ba.

All the constructed plasmids were confirmed by restricted digestion and sequencing. E. coli BL21(DE3) cells were transformed with each recombinant plasmid, and protein expression was induced with IPTG and analyzed by SDS-PAGE. All the expressed proteins were predominantly found in inclusion bodies. The expressed quantity of B-cell epitopes rE2-a, rE2-b and rE2-ba were about 41%, 40%, and 38% of total expressed proteins in E.coli, respectively, by densitometric scanning (Figure 2A). Complete solubilization of the inclusion bodies was achieved in urea buffer. Then, these epitope proteins were purified by Ni^2+^-NTA resin under denaturing conditions and eluted in elution buffer. After SDS-PAGE and Coomassie brilliant blue staining, three purified proteins displayed a single band with a molecular weight of 22 kDa, 22 kDa and 25 kDa, respectively. The results of Western blot assay indicated that purified rE2-a, rE2-b and rE2-ba were specifically recognized and strongly reacted by CSFV positive sera (Figure 2B). It suggested that the three linear peptides all possessed antigenicity.

### 3.2 Cell-mediated immune response to CSFV in vaccinated pigs

**Figure 3:**
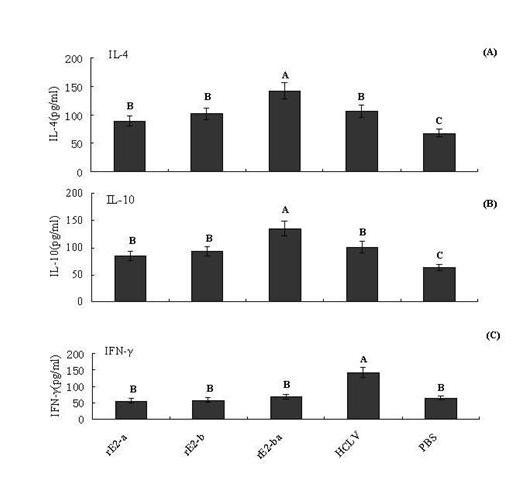
**Cytokine response to vaccination**. (A) IL-4 (pg/ml); (B) IL-10 (pg/ml); (C) IFN-γ (pg/ml) expressed as the mean ± standard error. Data shown are representative of three separate experiments. Statistically significant differences (p < 0.05) are indicated with different letters.

Cellular immune response was evaluated by the production of IFN-γ, IL-4 and IL-10 induced by all vaccinated pigs. As shown in Figure 3, the levels of IL-4 and IL-10 induced by rE2-ba were significantly higher than that of any other groups (P < 0.05), no significant differences were noted in the production of IL-4 and IL-10 in pigs immunized with HCLV vaccine, rE2-b or rE2-a. The IFN-γ levels were low but uniformity stably in three peptide vaccines, while HCLV could induce the high level of IFN-γ(P < 0.05). The levels of Th2 cytokines response were affected in pigs vaccinated peptide vaccines, albeit to differing degrees, but Th1 cytokines response could not occur. Therefore, MEV could stimulate pigs to induce intense humoral immune response.

### 3.3 Antibody response in vaccinated pigs

all immunized pigs were bled weekly following primary vaccination for determining neutralizing antibody(NA) production induced by these epitope peptides in vitro by NPLA assay. When the second boost vaccination, NA begined to emerge in all of pigs immunized with MEV or HCLV(positive controls). In the 1st week after viral challenge, the NA titers were the most highest peak in pigs vaccinated with peptide vaccine or HCLV and NA titer of pigs induced by rE2-ba was much higher than that induced by either mono-epitope peptide vaccine(rE2-a or rE2-b) or HCLV(p < 0.05). No neutralization activity against CSFV was determinated with serum from PBS-immunized pigs in the whole experiment (Table 3). These data implied that the humoral immune response induced by the multi-epitope was better than that induced by the mono-epitope.

**Table 3:** Pigs immunized with rE2-a, rE2-b and rE2-ba elicited serum neutralizing antibodies early after viral challenge.

Groups	Pigs	Inoculum	Neutralizing antibody titer									
			
			**day p.i**.					**day p.c**.				
			
			**0 **^**a**^	7	**14 **^**b**^	21	28	**35**^**c**^	42(7)	49(14)	56(21)	63(28)
A	5	rE2-a	< 2	< 2	4	16	32	32	64	64	64	64
B	5	rE2-b	< 2	< 2	8	32	64	64	128	128	128	128
C	5	rE2-ba	< 2	< 2	8	32	64	64	256	256	256	256
D	5	HCLV	< 2	< 2	8	16	32	64	128	128	128	128
E	5	PBS	< 2	< 2	< 2	< 2	< 2	< 2	< 2	< 2	< 2	0^d^

Antibody titers were determined using a NPLA assay, as described in the study. Samples were collected every 7 days. a, first innoculation; b, second innoculation; c, CSFV strain Shimen challenge timepoint. d, all of the pigs in the group E had died after 28 dpc.

### 3.4 Clinical signs and virus detection after CSFV challenge infection

**Figure 4:**
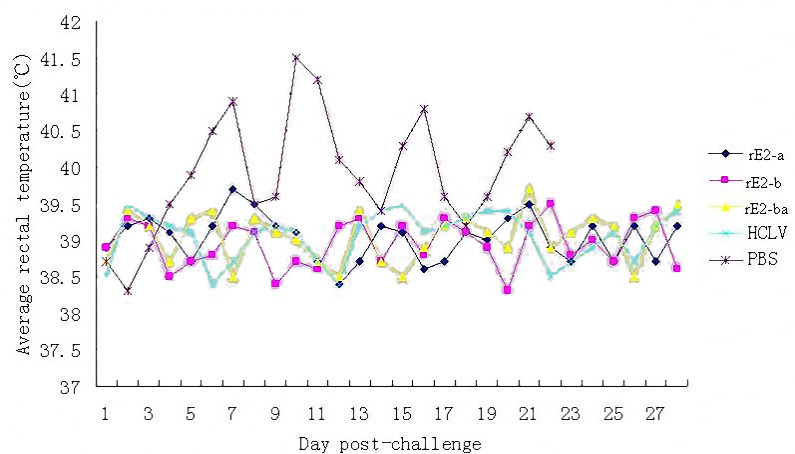
**Body temperature change (average body temperature curves of each group) of pigs after post-challenge of CSFV Shimen strain**. Body temperature was measured every day. Symbol (◆) represents the average body temperature of Group A (immunized with rE2-a). Symbol (■) represents the average body temperature of Group B (immunized with rE2-b). Symbol (▲) represents the average body temperature of Group C (immunized with rE2-ba). Symbol (×) represents the average body temperature of Group D (immunized with HCLV). Symbol (*) represents the average body temperature of Group E (immunized with PBS). Obviously, the average body temperature of Groups A, B and C waves in the normal body temperature range (39.0 ± 0.5°C) after post-challenge (dpi with CSFV Shimen strain). In contrast, the average body temperature of Group E in the same period is 1~2°C higher. All pigs immunized with PBS died during the period from 14 to 21 dpi.

In the 2nd week after lethal CSFV challenge, three groups of pigs immunized with rE2-ba, rE2-b and HCLV vaccine returned to health, as before viral challenge, which corresponded to a 100% protection rate. However, pigs immunized with rE2-a only induced 80% protection and remained infected (Table 4). The average body temperature of these four groups was in the normal range after viral challenge (Figure 4), while all PBS-immunized pigs in negative control group manifested obvious typical symptoms and died during the second and third week after viral challenge (Table 4). On postmortem examination only the PBS-immunized control and one of the rE2-a- immunized pigs showed petechiae on the kidney. No other pathological changes were observed

**Table 4:** Protective potency of the expressed peptide vaccine against lethal challenge by CSFV Shimen strain.

Group	Vaccination	Clinical sign			Morbidity (%)	Mortality (%)	Protection Ratio (%)
					
		Petechial heamorrhages	Diarrhea	Endothelial damage			
A	rE2-a	1/5	1/5	1/5	20	20	80
B	rE2-b	0/5	0/5	0/5	0	0	100
C	rE2-ba	0/5	0/5	0/5	0	0	100
D	HCLV	0/5	0/5	0/5	0	0	100
E	PBS	5/5	5/5	5/5	100	100	0

macroscopically. Samples of spleen tissue were examined for CSFV in Cell inoculation as well as in diagnostic Q-PCR described by zhou *et al* [29]. CSFV could be detected in the spleens of all the PBS-vaccinated and one of the rE2-a-immunized pigs in both assays. These results demonstrated that the multiple epitope rE2-ba could completely protecte pigs from lethal viral challenge, similar to the conventional HCLV vaccine, and suggest a new way to develop a epitope-based vaccine against CSFV.

## Discussion

Epitope-based vaccine is one of the promising vaccine development strategies in the prevention of variant diseases or against the different stages of some diseases. It has serveral advantages over the conventional vaccine, such as safety, low cost, multivalence, efficient antigen presentation, and ease of application. On base of CSFV glycoprotein E2, Many epitopes have been investigated by monoclonal antibodies, phage display technique or polypeptide synthesis.

TAVSPTTLR(aa 829-837) was identified as a CSFV-specific linear epitope of CSFV E2 protein, but did not characterize whether the epitope can induce protective immune response [20]. Based on this core sequence, a fusion protein was expressed to inject into pigs, but did not produce complete protection [18], only two of five vaccinated piglet produced significant antibody level and survived the challenge. These results indicated that the fusion protein containing TAVSPTTLR epitope sequence has a certain neutralizing activity, though it is not complete. Recently the protective immunity of two major epitopes on CSFV E2 had been investigated [21,23]. The data showed that synthetic peptide aa 693-716 (rE2-b) on CSFV E2 could induce the complete protection in pigs against a lethal challenge of CSFV, while synthetic peptide aa 844-865 (rE2-a) could only induce the incomplete protection. In our study, three peptides were expressed respectively by a prokaryotic expression system and the antibody response induced by multi-epitope peptide rE2-ba and mono-epitope peptide rE2-b or rE2-a were investigated *in vivo*. The results indicated that all three proteins could elicit antibody responses, with rE2-ba inducing the greatest response, similar to that of HCLV vaccine.

Prokaryotic expression systems show high expression levels, especially for brachy-peptide such as linear B-cell epitopes [30]. The three peptides in the present study were short peptides, therefore, which were expressed in a prokaryotic system induced by IPTG, and a high expression of about 40% of total protein was obtained. It found that more soluble protein was expressed on the condition of 20~30°C. Accordingly, these expressed proteins mought replace synthetic peptides and be mass produced as a potential DIVA (differentiating infected from vaccinated animals) vaccine for large numbers of pigs.

These linear B-cell epitope peptides were expressed with the form of a fusion protein, combined with ISA 206 VG adjuvant, and injected into pigs. They elicited a high level of humoral immunity by NPLA assays. Neutralizing antibodies in pigs immunized with purified proteins were determined before and after viral challenge. Meanwhile, three weeks after the second immunization, the antibody titers of all pigs inoculated with rE2-ba and rE2-b reached about 50% blocking rate by IDEXX HERDCHEK* CSFV Antibody Kit (data not shown). It suggested that antibodies induced by rE2-ba and rE2-b provided adequate protection against CSFV. In contrast, the antibody titers in a few pigs inoculated with rE2-a did not exceed 40% blocking rate(data not shown), which indicated that rE2-a provided partial immune protection.

To understand the cytokine response to these linear B-cell epitopes, several cytokine levels were estimated by ELISA. The results showed that the three epitope peptides led to the following changes. HCLV contained both T-cell and B-cell epitopes that induced Th1 and Th2 responses in vivo, so the concentration of IFN-γ induced by the three expressed peptide vaccines was lower than that in the pigs immunized with HCLV. In contrast, B-cell epitope peptides induced mainly Th2-type cytokine production. Therefore, the levels of IL-4 and IL-10 were more higher in pigs immunized with these three peptides.

In conclusion, the immunogenic and especially protective properties of the rE2-ba protein as multiple epitope vaccine were tested under lethal challenge conditions in pigs. Furthermore, CSFV-specific neutralizing antibody formation as well as the IL4/10-secreted were induced by the E.coli-expressed epitopes, the complete protection was observed. It suggest that rE2-ba should be developed as a new candidate epitope-based CSFV vaccine, by combination with two or more epitopes.

## Competing interests

The authors declare that they have no competing interests.

## Authors' contributions

BZ and KL carried out most of the experiments and wrote the manuscript. YJ and JCW helped with the experiments. PYC designed the experiments and revised the manuscript. All of the authors read and approved the final version of this manuscript.
